# The vector ecology of introduced *Culex quinquefasciatus* populations, and implications for future risk of West Nile virus emergence in the Galápagos archipelago

**DOI:** 10.1111/mve.12329

**Published:** 2018-08-31

**Authors:** G. Eastwood, A. A. Cunningham, L. D. Kramer, S. J. Goodman

**Affiliations:** ^1^ School of Biology University of Leeds Leeds U.K.; ^2^ Wildlife Epidemiology, Institute of Zoology Zoological Society of London London U.K.; ^3^ Galápagos Genetics Epidemiology and Pathology Laboratory Santa Cruz Ecuador; ^4^ New York State Department of Health Wadsworth Center New York NY U.S.A.

**Keywords:** disease ecology, invasive species, mosquito biology, vector‐borne disease, West Nile virus, Galápagos Islands

## Abstract

*Culex quinquefasciatus* Say (Diptera: Culicidae), an important vector of West Nile virus (WNV) in the U.S.A., was first detected on the Galápagos Islands (Ecuador) in the 1980s. However, little is known of its ecology, distribution or capacity for arbovirus transmission in the Galápagos. We characterize details of lifecycle (including gonotrophic period), temporal abundance, spatial distribution, vector competence and host‐feeding behaviour. *Culex quinquefasciatus* was detected on five islands of the Galápagos during 2006–2011. A period of 7–14 days was required for egg–adult emergence; water salinity above 5 ppt was demonstrated to hinder larval development. Blood‐meal analysis indicated feeding on reptiles, birds and mammals. Assessment of WNV vector competency of Galápagos *C. quinquefasciatus* showed a median infectious dose of 7.41 log_10_ plaque‐forming units per millilitre and evidence of vertical transmission (minimal filial infection rate of 3.7 per 1000 progeny). The distribution of *C. quinquefasciatus* across the archipelago could be limited by salt intolerance, and its abundance constrained by high temperatures. Feeding behaviour indicates potential to act as a bridge vector for transmission of pathogens across multiple taxa. Vertical transmission is a potential persistence mechanism for WNV on Galápagos. Together, our results can be used for epidemiological assessments of WNV and target vector control, should this pathogen reach the Galápagos Islands.

## Graphical abstract

Invasive mosquito species can transmit novel disease pathogens; *Culex quinquefasciatus* Say is a recent invader of the Galápagos archipelago, where it has been found on five of the islands.
In Galápagos, high temperatures limit *C. quinquefasciatus* abundance, and water salinity hinders larval development. They blood‐feed on reptiles, birds and mammals, and could act as bridge vectors for arboviral transmission.
Experimental infection studies indicate Galápagos *C. quinquefasciatus* can transmit West Nile virus vertically to progeny, as well as horizontally to potential susceptible hosts.

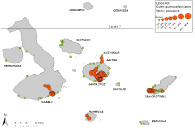

## Introduction

Assessing the ecology of hosts and vectors involved in the transmission cycles of infectious pathogens is key to understanding the potential epidemiology and critical factors affecting disease maintenance and transmission (Institute of Medicine (U.S.) Forum on Microbial Threats, 2008). Emerging infectious diseases are widely recognized as impacting public and animal health (Jones *et al*., 2008). In addition, they can compromise ecosystem function and threaten species of conservation concern (Daszak *et al*., 2000; Maillard & Gonzalez, 2006). New or re‐emerging pathogens, including those with expanding geographical or host ranges, can adversely affect populations of novel hosts, act as selective forces on host evolution, upset ecosystem balance or have serious consequences for species survival (Altizer *et al.,* 2003; Smith *et al.,* 2006). For example, the chytrid fungus *Batrachochytrium dendrobatidis* Longcore, Pessier & D.K. Nichols (Rhizophydiales) is causing population extirpations of amphibian species globally (Skerratt *et al*., 2007), while sylvatic plague nearly drove endangered black‐footed ferrets to extinction in North America by depleting their prey base (Daszak *et al*., 2000; Biggins & Godbey, 2003). When introduced to an immunologically naive population, a pathogen can be devastating even for a common and widespread species, such as occurred with finch trichomonosis in the U.K. and the dramatic emergence of West Nile virus (WNV) in the U.S.A. (LaDeau *et al*., 2007; Robinson *et al.,* 2010). Island populations, which are often small and naive to many pathogens, are particularly vulnerable to emerging infectious diseases; a classic example is the extinction in Hawaii of birds susceptible to introduced avian malaria (van Riper *et al*., 1986). In addition to birds, WNV (a mosquito‐borne *Flavivirus*) can kill reptiles and mammals (Kramer *et al*., 2008), and following its spread into Central and South America the virus now poses a potential threat to the endemic reptilian and avian fauna of Galápagos (Kilpatrick *et al*., 2006a; Eastwood *et al*., 2014).

Historically, the Galápagos archipelago, located 1000 km off the coast of Ecuador, has been an isolated ecosystem. The need for improved biosecurity for Galápagos, to reduce the risk of incursion of WNV and other novel pathogens that could threaten this UNESCO World Heritage site, has previously been highlighted (Wikelski *et al*., 2004; Kilpatrick *et al*., 2006a; Deem *et al*., 2012; Eastwood *et al*., 2013). Understanding the drivers and pathways of pathogen incursion and mechanisms of local spread and persistence is key to developing protocols for disease prevention and control. Such risk analyses and preparedness also improve the ability to detect pathogen incursion early and to respond rapidly and effectively should this occur. For arthropod‐borne pathogens, it is necessary to determine both the ecology of potential vector(s) and their ability to transmit the pathogen in question.

Vectoral capacity (which includes several parameters, including biting rates, probabilities of transmission or infection per bite, and vector survival) addresses the inherent host, virus and vector interactions for a vector to facilitate disease spread (Liu‐Helmersson *et al*., 2014). The primary traits that determine a mosquito species' role as a WNV vector include vector competence (the ability to be infected with, disseminate and transmit a pathogen) and host‐feeding preferences, while high abundance and wide distribution promote the likelihood of disease emergence (Kramer & Kilpatrick, 2010). It has been shown that an endemic Galápagos lineage of the black salt marsh mosquito, *Aedes taeniorhynchus* (Wiedemann) (Bataille *et al*., 2009b), is a competent WNV vector and both abundant and widespread across the archipelago (Eastwood *et al*., 2013). However, Galápagos *A. taeniorhynchus* appears to have a low feeding rate on birds, suggesting that it might not be a primary concern for the establishment of WNV on Galápagos (Eastwood *et al*., 2013). Conversely, *Culex quinquefasciatus* Say might be a more important candidate vector since the species is a key vector of WNV in the southern U.S.A. (Kramer *et al*., 2008). *Culex quinquefasciatus* was first detected in Galápagos in 1989 (Peck *et al*., 1998; Whiteman *et al*., 2005), and genetic evidence suggests there have since been ongoing introductions, and human‐mediated transport across the archipelago (Bataille *et al*., 2009a). Little is known about the extent of *C. quinquefasciatus* distribution outside urban areas, and we hypothesize it has potential to spread more widely through the islands. Similarly, the ecology of *C. quinquefasciatus* on Galápagos, or how this might influence vectorial capacity for mosquito‐borne pathogens, is not well understood. Although we have already shown Galápagos *C. quinquefasciatus* to be a moderately competent and efficient vector of WNV (Eastwood *et al*., 2011), the role of this mosquito in the epidemiology of WNV (or other pathogens) will depend on a range of additional factors, such as abundance, distribution, life‐stage durations, host utilization, gonotrophic cycle length and tolerance to different environmental conditions. Here, we describe these ecological characteristics for *C. quinquefasciatus* in Galápagos and assess its predicted vector capacity and role as a vector of WNV on the archipelago. Such ecological data can provide valuable information for conservation managers, entomologists and public health authorities seeking to predict and mitigate the role of *C. quinquefasciatus* in disease transmission in the contexts of human health, agriculture and conservation.

## Materials and methods

To characterize the ecology of *C. quinquefasciatus* on Galápagos, we aimed to determine life‐stage durations, spatial distribution, temporal abundance and host‐feeding patterns, as well as further aspects of this mosquito's interaction with WNV (infectious dose, vertical transmission).

### *Distribution and abundance*

*Culex quinquefasciatus* was collected as part of a multi‐year mosquito‐monitoring programme as described Eastwood *et al*. (2013), during which U.S. Centers for Disease Control (CDC) light‐trap abundance data were obtained across nine Galápagos islands from 2006 to 2011, along with presence–absence data being recorded using CDC gravid and BG Sentinel traps (John W Hock Co., Gainesville, FL, U.S.A.) (Eastwood *et al*., 2013). Traps were set in eight different vegetation zones of Galápagos, including urban areas, and the *C. quinquefasciatus* were identified morphologically, with the species assignment having previously been confirmed via microsatellite profiling (Bataille *et al*., 2009a). These data were similarly mapped geospatially over a digital elevation model of the Galápagos Islands, and determinants of mosquito abundance were assessed using detections from Santa Cruz Island (where more records were available), with predictive factors and modelling (with backward selection) as described by Eastwood *et al*. (2013).

### *Development stages of immature mosquitoes*

Galápagos *C. quinquefasciatus* were captured using four CDC gravid traps, or oviposition tubs (dark‐coloured plastic pots filled with water) to collect gravid females or eggs respectively. Both trap types were employed overnight in Puerto Ayora during November 2010 and used an attractant of water infused with local cut‐grass for 5 days. Captured gravid female mosquitoes were transferred to 30 × 30 × 30 cm^3^ Bugdorm™ cages (MegaView Science Co., Ltd, Taiwan) and supplied with an oviposition cup to encourage egg laying and were fed *ad libitum* with 10% sucrose solution. Egg rafts (*n* = 6 replicates) were transferred individually to new containers of water with salinity of approximately 2.9 parts per thousand (ppt), and development through each life stage was monitored. The duration that immature mosquitoes spent as (a) eggs, (b) larval stages and (c) pupae was recorded to the nearest 6 h. Rearing conditions matched typical Galápagos climate, averaging 26 °C with a relative humidity (RH) of 80% and 12 : 12 h light : dark (LD) photoperiodicity. Following eclosion from eggs, the mosquito larvae were held in groups in plastic containers (25 × 30 × 5 cm^3^) in water (approximately 1 L per 100 larvae) and provided with ground Koi fish food (Tetra®, VA, U.S.A.) (roughly 3 mL per container per day). Pupae were transferred to emergence jars (Bioquip Products Inc., Compton, CA, U.S.A.). Emerged adults were transferred to Bugdorm cages (MegaView Science Co., Ltd) and used for longevity investigations. The period of each life stage was timed for 742 individuals from the six egg rafts.

### *Developmental tolerance to salinity*

The effect of salinity on mosquito development was monitored by placing egg rafts, obtained as noted earlier, in individual pots, five per treatment, containing 0.5 L of water with salt concentrations ranging from 0 to 15 ppt in 1 ppt intervals, 20 ppt and 25 ppt, and the concentration at which hatching and development failed was determined. Solutions were prepared using fresh water or by successive dilutions of brackish or seawater (21 ppt) for the 0–20 ppt groups, with further salt (Instant Ocean®, U.S.A.) added to obtain a solution of 25 ppt Salinity was measured using an EC300 conductivity meter (YSI, Yellow Springs, OH, U.S.A.). Food was supplied *ad libitum* (approximately 1 mL per pot per day) until pupal eclosion or death occurred. Development status was recorded daily, with any dead individuals noted and removed. The proportion of each egg‐raft hatching was estimated visually to the nearest 5% based on original size of raft remaining. Specimens were maintained in flats or emergence jars according to development stage.

### *Longevity*

Adults (*n* = 183) emerging within a 24 h time period from the six egg rafts in the first experiment (each from a different female) were held together in Bugdorm cages, inside but open to the Galápagos climatic conditions as described earlier. A bloodmeal of heparinized chicken blood was provided at 96 h post‐emergence, being presented overnight within a sausage casing membrane (Natural Sausage Skins, Cirencester, U.K.). Blood‐fed mosquitoes were retained from those not having fed, and oviposition was allowed through the provision of oviposition cups. Natural conditions were simulated by mixing both sexes and allowing oviposition, as virginity and lack of blood feeding may extend longevity (Gunay *et al*., 2010). Individuals were held, supplied with 10% sucrose *ad libitum*, and monitored until death. Each dead mosquito *i* was then removed from the cage, sexed and the time *t*_*i*_ recorded since the first adult emergence. A tally of survival data for the female mosquitoes was recorded, enabling the range of longevity and mean durations of adult life to be determined. Survivorship was considered as a percentage of the group of emerged mosquitoes alive at time *+t* days.

### *Host‐feeding behaviour*

Engorged mosquitoes were collected using resting traps or CDC light traps, from sites on Santa Cruz, Isabela, Baltra and San Cristobal islands in 2009 and 2010, as described previously (Eastwood *et al*., 2013). DNA was extracted from the abdomen using a Chelex protocol, followed by polymerase chain reaction assays to amplify the vertebrate mitochondrial cytochrome b (*Cyt b*) gene for sequencing as described by Eastwood *et al*. (2013). The host on which the mosquito had fed was determined by comparison of sequences to those available on Genbank using blast (https://blast.ncbi.nlm.nih.gov).

### *Gonotrophic cycle*

The length of the gonotrophic cycle was recorded as the time between a mosquito taking a bloodmeal and the deposition of eggs. Five groups of 25 matured and mated female *C. quinquefasciatus* were fed chicken blood overnight via a sausage skin membrane. Engorged mosquitoes were moved to a separate cage and held at either 26 °C (three groups) or 29 °C (two groups), with an LD 12 : 12 h photoperiod and 80% RH; all provided with sucrose solution *ad libitum* and an oviposition cup for egg deposition. Bloodmeals infected with WNV (7.46 log_10_ plaque‐forming units [PFU]/mL of strain WNV02‐1956) were presented to one group at each temperature to investigate whether the presence of the virus had any effect on the gonotrophic cycle. Mosquitoes were subsequently held in their respective groups, as noted earlier. The period of time from each feeding (day 0) until egg deposition was recorded. Deposited eggs were continually removed from the oviposition cup to enable monitoring of remaining gravid females, up to day 15 when the experiment was ended.

### *Determination of WNV infectious dose*

In order to determine the median infectious dose (ID_50_, i.e. the dose that would infect 50% of the mosquitoes) for the species, two populations (field‐collected Galápagos *C. quinquefasciatus* [*n* = 450] and a colony of U.S. *C. quinquefasciatus* [*n* = 150]) were each exposed to three doses of WNV (i.e. six total treatment combinations). The different doses of WNV (strain WNV02‐1956), subsequently confirmed by titre assay (Payne *et al*., 2006) to be 8.44 log_10_ PFU/mL (high dose), 7.46 log_10_ PFU/mL (medium dose) or 6.22 log_10_ PFU/mL (low dose), were presented to mosquitoes within a (defibrinated) goose bloodmeal held in a sausage membrane (Eastwood *et al*., 2011, 2013). Mosquitoes were subsequently held, in respective groups, with access to 10% sucrose solution *ad libitum*. Conditions were 25–27 °C with an LD 12 : 12 h photoperiod and 80% RH. All mosquitoes were tested for evidence of WNV infection at 10 days post‐exposure using a plaque assay (Eastwood *et al*., 2011) to determine WNV infection rates for each *C. quinquefasciatus* lineage (Galápagos vs. U.S.A.) and the influence of viral dose.

### *Vertical transmission*

Female *C. quinquefasciatus* (both field collected in the Galápagos [*n* = 120] and a control group of colonized U.S. *C. quinquefasciatus* [*n* = 70]) were infected with WNV (as earlier) using 8.44 log_10_ PFU/mL (the same bloodmeal preparation was divided between mosquitoes of each origin) and then maintained as in the previous section. At 4 days after egg rafts were deposited, parent mosquitoes were fed a further (uninfected) bloodmeal to promote oogenesis for subsequent oviposition cycles (OV_*i*_). Egg rafts were allowed to hatch and were reared under the conditions described earlier, then screened for WNV as fourth instar larvae. Offspring of each oviposition cycle (OV_1_, OV_2_, OV_3_) from single females were triturated as individual pools of 20–40 larvae in microfuge tubes containing 800 μL diluent (phosphate‐buffered saline, 20% fetal bovine serum and antibiotics). RNA was extracted from each supernatant using a QIAamp Viral RNA Mini kit (Qiagen Inc., Valencia, CA, U.S.A.) according to the manufacturer's instructions. The extracted RNA was tested for the presence of WNV RNA using real‐time reverse‐transcriptase polymerase chain reaction (as described by Eastwood *et al*., 2011).

### *Transmission titre*

To determine the WNV titre that Galápagos *C. quinquefasciatus* can inoculate when biting hosts, 63 salivary secretion samples, obtained as described by Eastwood *et al*. (2011), from mosquitoes that had been exposed to WNV during that study, were titrated by plaque assay, as described by Payne *et al*. (2006). These mosquitoes had experimentally been fed bloodmeals containing WNV (median titre 7.7 log_10_ PFU/mL), then held at nine different combinations of temperature (27 °C or 30 °C) and extrinsic incubation period (EIP) post‐infection (days 7, 11, 14, 21 or 28; *n* = 18 mosquitoes per time‐point, except survival prevented testing on day 28 at 30 °C) prior to harvesting (Eastwood *et al*., 2011); samples had been stored at −80 °C until processing.

### *Statistical analysis*

Distribution and abundance data were analysed as described previously, using a zero‐inflated generalized linear model (GLM) with negative binomial error structure, to assess environmental and ecological determinants of mosquito abundance for Santa Cruz Island (where most records were available) as described by Eastwood *et al*. (2013). Highland and coastal abundance was compared using a *t*‐test, and difference between years using a *Z*‐test. Average life‐stage development durations were calculated using mean and standard deviation. As well as calculating the mean of gonotrophic cycle length per group, *t*‐tests were performed to compare the effect of temperature, and that of WNV exposure. Chi‐square tests were used to assess site differences in host utilization. Mean longevity within egg batches was calculated by multiplying survival time *t* by the number of mosquitoes living until time *t*, then dividing the sum of each product by the total number of mosquitoes in the egg batch. Survival analysis and confidence intervals for median lifespan were derived using r software and the r package ‘survival’ (R Core Team, 2012).

Infection rates were calculated as the percentage of all mosquito bodies tested in that group from which WNV was detected. Infectious doses of WNV that would infect 50% of the mosquitoes (ID_50_) were calculated according to the Reed–Muench formula index (Reed & Muench, 1938). The ID_50_ of U.S. and Galápagos lineages of *C. quinquefasciatus* were compared using 95% binomial confidence intervals for a difference, based on the standard error of the numerator *E*(*x*) and denominator *E*(*y*) of Eq. 1; *α* = 0.05 level of significance was used to construct confidence intervals:

(1) $$\begin{array}{cc} & {\mathrm{ID}}_{50}\left(\mathrm{Gal}\overset{´}{a}\text{pagos}\right)-{\mathrm{ID}}_{50}\left(U.S.\right)\\ & \pm z_{a/2}\sqrt{(\mathrm{Var}\left(\mathrm{Gal}\overset{´}{a}\text{pagos}\text{\_}\text{Index}\right)+\mathrm{Var}\left(U.S.\text{\_}\text{Index}\right)} \end{array}$$

where the variance of each group was calculated as the variance of the ratio of two random variables:

$${\left(\frac{E\left(x\right)}{E\left(y\right)}\right)}^{2}\times \left(\frac{\mathrm{Var}\left(X\right)}{E{\left(x\right)}^{2}}+\frac{\mathrm{Var}\left(Y\right)}{E{\left(y\right)}^{2}}-\frac{\mathrm{Cov}\left(x,y\right)}{E\left(x\right)E\left(y\right)}\right)$$

The vertical transmission rate of WNV in *C. quinquefasciatus* was determined as the percentage of infected females that transmitted virus to their progeny, regardless of the infection rate in the progeny (Turell, 2008). The minimal filial infection rate (MFIR) refers to the minimum number of F_1_ mosquitoes vertically infected with WNV per 1000 progeny. MFIR was based on one infected mosquito per group of pooled mosquitoes, calculated as the number of WNV‐infected larvae pools divided by the total number of larvae tested, multiplied by 1000.

Salivary secretion titres were log‐transformed (log_10_ PFU/secretion sample) and adjusted for total dilution (1 : 1000) in diluent and inoculum. The effect of temperature upon WNV titres was assessed using a *t*‐test (at a 95% level of significance) to compare groups, and the effect of EIP using anova. All statistics were calculated using r.

## Results

### *Distribution and abundance*

**Figure 1:**
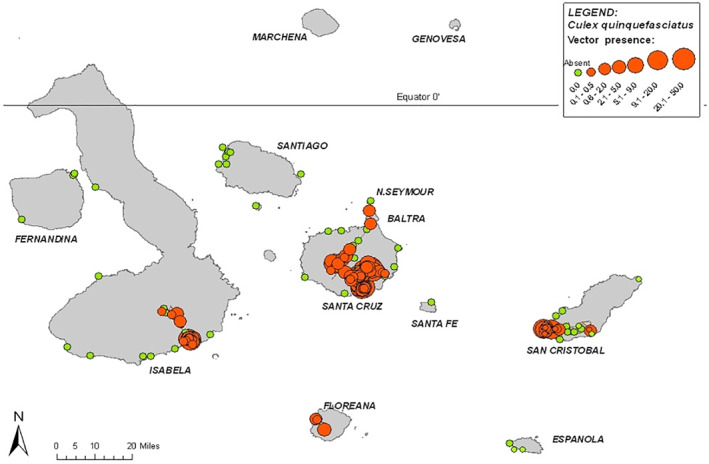
Presence of *Culex quinquefasciatus* at 127 sampled locations in Galápagos (circle size indicates relative abundance). [Colour figure can be viewed at wileyonlinelibrary.com].

A total of 1403 trap‐nights were made across 127 sites on nine Galápagos islands, yielding 5241 *C. quinquefasciatus*. During the 5 years of monitoring, the species was detected on the inhabited islands of Baltra, San Cristobal, Santa Cruz, Floreana and Isabela, but not on the uninhabited islands of Santiago, Española, Rabida or Santa Fé (Fig. 1). Sampling at these latter uninhabited islands took place less frequently due to logistical constraints. In Santa Cruz, *C. quinquefasciatus* was found during sampling in the highlands and in the northerly, uninhabited, part of the island, as well as in the villages on the south side of the island. Detections were also made in the highlands of Isabela and San Cristobal. There was no difference in mean abundance between highland and coastal sites (Welch *t*‐test: *t* = 1.77, d.f. = 1016, *P* = 0.96). However, abundance increased significantly year on year (*Z* = −19.2, *P* < 0.001), with the exception of 2009 when fewer mosquitoes were observed in total during the monitoring period. Spatial, temporal and abiotic factors, including temperature, precipitation (and lagged variables thereof), distance to sea, distance to urbanization (nearest human dwellings according to geographic information system and ground truthing), vegetation zone, latitude, longitude, elevation, year, month, tide height and moon phase were fitted as model terms, with all except the last three being significant as individual predictors of abundance (*P* < 0.001–0.05; Table 1), along with interaction terms. Since abundance did not have a linear relationship with temperature, polynomial regressions were applied to the covariate describing ‘temperature’. Coefficients of the zero‐inflated model are displayed in Table 1. This model was found to be more appropriate than a standard negative binomial GLM (Vuong test‐statistic *V* = 1.92, *P =* 0.028). There was minimal spatial pattern in the abundance model (seen in a variogram of model residuals and confirmed by a geospatial fit to the model using latitudinal and longitudinal co‐ordinates: range, 0.066; partial sill, 1.15; nugget, 0.73).

**Table 1:** Summary of determinants of Galápagos *Culex quinquefasciatus* (female) abundance, using a negative binomial generalized linear model (GLM) model (on count data) with a zero‐inflated component (explaining factors contributing to zero abundance).

Zero‐inflated GLM (Neg. binomial with logit link)		Predictor	Coeff.	Std. error	*Z*‐value	*P*
Galápagos *Culex quinquefasciatus* abundance log(*θ*) = −1.96 (*P* < 0.001***) Log‐likelihood = −966.4 (d.f. = 11)		Intercept	6.35	1.28	4.96	<0.001***
	Vegetation zone (χ^2^ = 91.4, *P* < 0.001) (agriculture default)	Arid	1.00	0.29	3.48	0.0005***
		Coast	−2.93	0.49	−5.96	<0.001***
		Mangrove	−0.66	0.31	−2.12	0.034*
		*Scalesia*/*Miconia*	−2.28	0.62	−3.66	<0.001***
		Transition	−0.74	0.51	−1.45	0.146
		Urban	−1.08	0.39	−2.73	0.006**
	Climate	Mean temperature (°C) (polynomial)	−0.25	0.05	−4.62	0.001**
	Zero inflation component	Intercept	−13.57	4.35	−3.12	0.002**
		(Log) distance (m) to urbanization	1.78	0.55	3.21	0.001**

**Figure 2:**
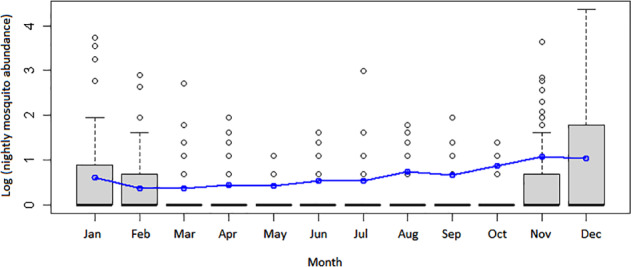
Plot of mean abundance of (female) *Culex quinquefasciatus* mosquitoes by month of year (based on Santa Cruz specimens). Line indicates model predictions. [Colour figure can be viewed at wileyonlinelibrary.com].

From the minimal adequate best‐fit model, significant predictors of *C. quinquefasciatus* in the Galápagos were vegetation zone, temperature and proximity to urbanization. Arid‐type vegetation zones resulted in a positive association with abundance, and *Scalesia* spp./*Miconia* spp. vegetation (cloud forest tree or shrub species endemic to Galápagos) a disassociation. Temperature had a negative effect on abundance, with sensitivity analysis showing each per‐unit increase in temperature to reduce log(abundance) by 0.25. Distance from urbanization (log) increased the chance that mosquitoes were absent, via the zero‐inflated portion of the GLM. Plotting abundance by month of the year (based on Santa Cruz collections, Fig. 2) showed that peak abundance of *C. quinquefasciatus* is predicted to occur between November and January.

### *Development stages*

A total of 731 adults emerged: 11 larvae died prior to pupation, and between 91 and 176 adults were produced from each egg raft. The mean time plus/minus standard deviation from egg deposition to egg eclosion was 1.22 ± 0.04 days (range 0.83–1.67 days). The mean total time from egg deposition to adult emergence was 10.61 ± 0.53 days (range 6.50–13.92 days), with individual stages averaging 8.23 ± 0.24 days (range 4.50–11.5 days) as larvae, and 1.15 ± 0.47 days (range 0.83–1.58 days) as pupae.

### *Tolerance to salinity*

**Figure 3:**
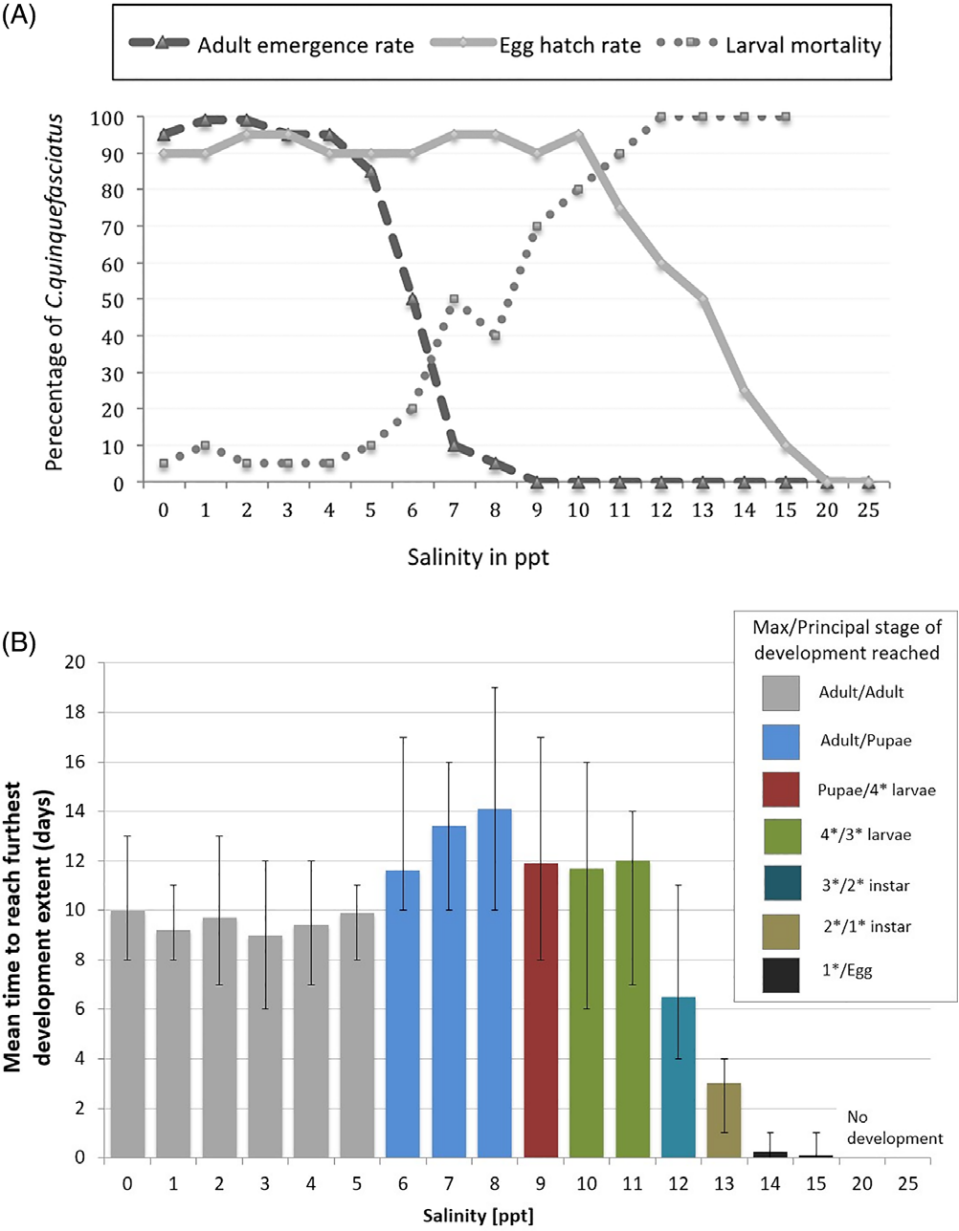
Effect of salinity (measured in parts per thousand) on the immature development of *Culex quinquefasciatus* in Galápagos. (A) Percentage of mosquitoes' eclosion or mortality rates. Hatch rate is the estimated proportion of the egg raft hatching to larvae. Larval mortality is the number of dead larvae (any instar stage) divided by total larvae. Emergence rate is the percentage of pupae becoming adult mosquitoes. (B) Time to development (days), from egg deposition until the predominant stage of development was reached (higher salinities limited survival, and for those cases the maximum extent of development is indicated). [Colour figure can be viewed at wileyonlinelibrary.com].

Mosquito emergence rates were high and larval mortality was low at salt concentrations below 5 ppt (Fig. 3A). Emergence rates dropped above 5 ppt, and maturation from egg to adult lengthened in duration (Fig. 3B). At 6 ppt, less than half of the initial larvae reached adult stage, and larval–pupal development took an average of 12 days. At 9 ppt, 70% of larvae died at or before the fourth instar, and the pupae that developed all failed to eclose to adults. Larvae were observed to turn pale in colour and slow in movement before death at this salinity. At 12 ppt, only the third (instar) phase of development was reached and there was 100% larval mortality. By 15 ppt, few larvae (estimated 10%) hatched from the egg raft and these all died as first instar larvae. No hatching occurred at salinities of 20 or 25 ppt.

### *Longevity*

**Figure 4:**
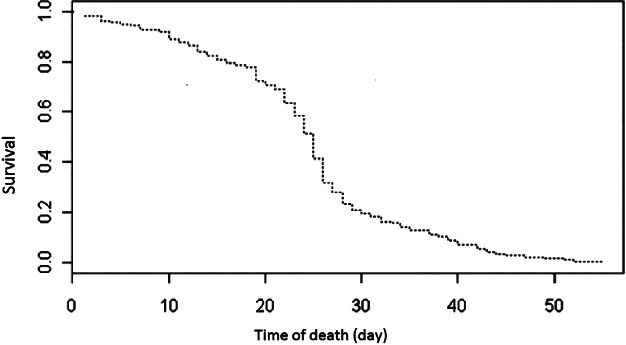
Species survivorship curve for *Culex quinquefasciatus* in Galápagos.

Adult survival ranged from 1 to 55 days, the mean being 24.03 days (standard deviation 9.66; median 25 days), with a Type I survivorship curve – higher survival probability at early/middle ages, followed by rapid decline in later life (Rauschert, 2010) (Fig. 4).

### *Host‐feeding*

Analysis of 69 bloodmeals (able to be sequenced from a total of 117 wild‐caught engorged mosquitoes) showed 75.4% to be mammalian, 20.3% to be avian and 4.3% to be reptilian (Fig. S1). There was no significant difference in the host choice of mosquitoes collected from highland sites and those collected from coastal sites (χ^2^ = 0.73 [with Yates' correction], d.f. = 2, *P* = 0.69). The most commonly bitten host was *Homo sapiens* Linnaeus (Primates: Hominidae) (human, 59.4%, *n* = 41). Other mammalian hosts were *Sus scrofa* Linnaeus (Artiodactyla: Suidae) (domestic pig or wild boar, 5.8%, *n* = 4), *Canis lupus familiaris* Linnaeus (Carnivora: Canidae) (domestic dog, 4.4%, *n* = 3), *Bos taurus* Linnaeus (Artiodactyla: Bovidae) (cow; 2.9%, *n* = 2) and *Rattus rattus* (Linnaeus) (Rodentia: Muridae) (black rat; *n* = 1). *Gallus gallus* (Linnaeus) (Galliformes: Phasianidae) (domestic chicken) and *Geospiza* spp. Gould (Passeriformes: Thraupidae) (Galápagos finch) each represented 7.3% (*n* = 5) of bloodmeals; additional avian hosts for *C. quinquefasciatus* were *Nyctanassa violacea* (Linnaeus) (Pelecaniformes: Ardeidae) (yellow‐crowned night heron; 2.9%, *n* = 2), *Myiarchus magnirostris* (Gould) (Passeriformes: Tyrannidae) (Galápagos flycatcher, *n* = 1) and *Dendroica petechia* (Linnaeus) (Passeriformes: Parulidae) (Galápagos yellow warbler, *n* = 1). Two bloodmeals from *Amblyrhynchus cristatus* Bell (Squamata: Iguanidae) (marine iguana) and one from *Microlophus albemarlensis* (Baur) (Squamata: Tropiduridae) (lava lizard) were also identified.

### *Gonotrophic cycle*

The gonotrophic period ranged from 3 to 11 days. At 26 °C the average time for oviposition was 5.4 ± 0.46 days, whereas at 29 °C this was 4.1 ± 0.35 days; the latter is significantly quicker (*t* = −4.3, d.f. = 58, *P* < 0.001). Allowing one additional day for the time that a mosquito would spend host‐seeking (Ahumada *et al*., 2004), averaged across both temperatures the overall average gonotrophic cycle for Galápagos *C. quinquefasciatus* is 5.95 ± 0.36 days. There was no difference in gonotrophic period for mosquitoes exposed to WNV compared with those not exposed to this virus (26 °C: group 1 vs. group 3 [*t* = 0.5, d.f. = 9, *P* = 0.3], group 2 vs. group 3 [*t* = 1.2, *P* = 0.6]; 29 °C: group 4 vs. group 5 [*t* = 1.5, *P =* 0.1]).

### *Infectious dose (WNV)*

A dose of 7.41 log_10_ PFU/mL was required to infect 50% of Galápagos *C. quinquefasciatus* with WNV, and 6.84 log_10_ PFU/mL for U.S. *C. quinquefasciatus*, based on an incubation period of 10 days. The 95% confidence interval for the difference of their interval (−0.295, 1.428) showed that these ID_50_ values of the two strains were not significantly different (since the interval contains zero). Details of the vector infection are presented in Table 2.

**Table 2:** Vector competence and ID_50_ summary.

Mosquito group	No. tested	WNV‐positive body	Infected (%)
Galápagos HIGH	31	21	67.7
Galápagos MEDIUM	27	14	51.9
Galápagos LOW	35	6	17.1
U.S. control HIGH	25	19	76.0
U.S. control MEDIUM	22	15	68.2
U.S. control LOW	18	7	38.9

All mosquitoes were exposed to West Nile virus (WNV) strain WNV02‐1956, held at 25–27 °C with 12 : 12 h photoperiod for 10 days.

### *Vertical transmission*

Eggs (OV_1_) were laid approximately 4 days post‐exposure to WNV. Fifty OV_1_‐pools of F_1_ Galápagos *C. quinquefasciatus* larvae were tested for the presence of WNV. No OV_1_ larval pools tested positive for WNV. U.S. *C. quinquefasciatus* were not tested at the OV_1_ stage. Deposition of OV_2_ egg rafts began 4 days after the second feeding; i.e. 13 days post‐infection with the original WNV bloodmeal. The U.S. OV_2_ (F_1_) larvae that emerged, tested as 10 pools, were all negative for WNV. Of 18 pools of Galápagos OV_2_ larvae, two pools tested positive for WNV. Each OV_2_ pool contained around 30 mosquitoes, i.e. approximately 540 OV_2_ progeny in 18 pools were tested and from these results an MFIR was calculated as 3.7 per 1000 progeny. The mean titre of the infected pools was 1.1 log_10_ PFU/mL. Exact parentage was unknown; however, 15 of 25 Galápagos F_0_ mosquitoes screened were WNV positive, with an average body titre of 6.81 log_10_ PFU/mL. Only one OV_3_ egg raft was laid by Galápagos *C. quinquefasciatus* (at 26 days post‐infection). This was tested in three pools (each of approximately 20 larvae); all were negative for WNV.

### *Transmissible titres*

Titres tested in the salivary secretions of 63 WNV‐infected Galápagos *C. quinquefasciatus* ranged from 2.32 to 5.48 log_10_ PFU (mean 4.13 log_10_ PFU). This was not affected by either the ambient temperature at which the mosquitoes had been experimentally incubated (*t* = 0.925, d.f. = 5, *P =* 0.39) nor by the EIP (*F* = 2.99, *P =* 0.127).

## Discussion

Understanding vector ecology is vital when assessing the potential for vector‐borne disease transmission and conducting epidemiological risk assessments (Kilpatrick *et al*., 2006a). Preventing pathogen incursion in the first place is the ideal approach to protect wildlife from novel diseases, avoiding both the difficulties of eradication once a pathogen has been introduced into a population, and the social and economic repercussions that can result from disease outbreaks (Sleeman & Gillin, 2012). Given the global significance of Galápagos fauna, it is imperative to understand threats from invasive pathogens and parasites. An invasive parasitic fly, *Philornis downsii*, currently threatens endangered Galápagos finch species (Fessel & Tebbich, 2002; O'Connor *et al*., 2010), and there is concern over the risk of pathogen spillover from domestic chickens into wild birds (Gottdenker *et al*., 2005; Soos *et al*., 2008; Deem *et al*., 2012), and from dogs into Galápagos pinniped populations (Brock *et al*., 2013).

The risk that WNV would pose to Galápagos fauna, should this pathogen reach the islands, depends on the temporal and spatial distributions of competent vectors, their abundances and feeding habits. The data presented here can be used to identify locations, periods of the year and environmental/ecological factors that could influence the risk of arbovirus establishment and transmission, and therefore can inform development of mitigation strategies for vector control.

*Culex* is the genus of mosquito most frequently associated with WNV transmission elsewhere (Clark *et al*., 2004). Although we considered the role *C. quinquefasciatus* could have in the emergence of WNV on the islands, this work is also relevant to other pathogens vectored by mosquitoes, such as other arboviruses, avipoxvirus and certain haemoparasites. *Culex quinquefasciatus* first arrived in Galápagos around 30 years ago (Peck *et al*., 1998). Since then, air and boat transport links appear to have facilitated multiple introductions from the continent, and dissemination amongst islands (Bataille *et al*., 2009a). The current study is the first systematic, longitudinal (5 year) survey for investigating mosquito abundance across multiple islands annually. Until recently, this species was thought to be restricted to areas of human habitation on Santa Cruz (southern), San Cristobal (western), Floreana (northwestern), Isabela (southeastern) and Baltra islands (Whiteman *et al*., 2005; Bataille *et al*., 2009a). Here, we found *C. quinquefasciatus* outside inhabited zones on the main islands for the first time. In our study period, 2006–2011, no detections were made on the uninhabited islands of Española, Fernandina, Rabida, Santa Fé and Santiago, however *C. quinquefasciatus* detections on Santiago in 2013 and 2014 have been reported recently (Asigau *et al*., 2017). Although our dataset is subject to some bias, in that several islands, such as North Seymour and Fernandina, could only be sampled infrequently (<10 occasions), we show that *C. quinquefasciatus* is distributed more widely across the Galápagos Islands than previously thought, including in national park areas and agricultural zones. Asigau *et al*. (2017) also report distributions of *C. quinquefasciatus* and *A*. *taeniorhynchus* on Isabela and Santa Cruz in 2012–2014, consistent with our current and earlier studies (Bataille *et al*., 2009a, 2009b, 2010, 2011; Eastwood *et al*., 2013). The relative roles of human‐mediated transport, natural dispersal and local adaptation in the speed of establishment across the islands remain to be fully understood and are difficult to quantify due to a lack of historical monitoring for the presence of this mosquito. However, population genetic data do suggest that inter‐island boat traffic has facilitated mosquito dispersal in the recent past (Bataille *et al*., 2009a; 2011).

That, until recently, *C. quinquefasciatus* has not been detected on uninhabited islands (Asigau *et al*., 2017) and was absent or rare away from human‐modified habitats implies there are constraints on its dispersal and establishment, possibly due to a lack of suitable (human‐mediated) invasion pathways or habitat. Although it is encouraging that we did not find *C. quinquefasciatus* at most uninhabited sites, many locations are regularly visited by tourist boats and, therefore, are exposed to a potential introduction pathway (Bataille *et al*., 2009a). The current absence of *C. quinquefasciatus* at these sites despite high levels of boat traffic suggests that this potential introduction pathway may not be sufficient for establishment, or that some human modification of the environment is also important. One of the most vital habitat constraints is likely to be the presence of persistent bodies of fresh water. We showed that water salinities above 5 ppt constrained larval development. In other species, salt tolerance is related to the presence of a salt‐secreting gland for osmo‐ or iono‐regulation (Clark *et al*., 2004), but there are no data on these traits for Galápagos *C. quinquefasciatus*. In Galápagos, outside of human‐modified environments, fresh water sources are either absent or ephemeral. Within human‐modified environments, fresh water sources include domestic water supplies, unintentional reservoirs for rainwater (e.g. within tyres and discarded plastic containers), sources for agricultural use or created by agricultural practices. Fresh water availability, therefore, appears to be critical to limiting the distribution and abundance of *C. quinquefasciatus* on Galápagos. In order to distinguish the effect of salinity, larval competition and genetic background, a future experiment examining larval development and survival would use an equal number of larvae gained from a combination of different egg raft sources. Equally, surveys of the common characteristics for Galápagos *C. quinquefasciatus* larval habitats, including the level of organic material necessary, would be worthwhile pursuing.

We found the annual abundance of *C. quinquefasciatus* peaked in December, i.e. prior to the rainy season commencing in January (and also prior to the abundance peak of the endemic mosquito species *A. taeniorhynchus*; Eastwood *et al*., 2013). This might be counterintuitive, since mosquito abundance might be expected to increase with precipitation, as it does for *A. taeniorhynchus* (Galardo *et al*., 2009; Eastwood *et al*., 2013). However, drought conditions elsewhere have been associated with increased number of blood‐fed *Culex* spp. and increased WNV infection rates compared with wetter, milder seasons (Johnson & Sukhdeo, 2013). In Hawaii, rainfall patterns do not appear to regulate the abundance of *C. quinquefasciatus* due to sufficient rainfall year round to maintain breeding pools (Ahumada *et al*., 2004). In Galápagos, there are strong seasonal rainfall patterns but, as *C. quinquefasciatus* appears to be almost completely reliant on human‐mediated fresh water bodies for breeding, there is no synchrony between rainfall and mosquito abundance patterns, and temperature was found a more pertinent factor.

A key element affecting the impact of a newly introduced vector‐borne pathogen is the host‐feeding behaviour of the vector(s) (Kilpatrick *et al*., 2005). Although not assessed here, seasonal differences and host factors, such as migration, aggregate grouping and defensive mechanisms in response to mosquito biting, are likely to influence host selection, blood‐feeding success and thus patterns of arboviral transmission (Kilpatrick *et al*., 2006b; Wheeler *et al*., 2009). Across its global geographical range, the host‐feeding preferences reported for *C. quinquefasciatus* differ considerably, with some populations showing a preference for birds and others for mammals (Tempelis *et al*., 1970; Zinser *et al*., 2004; Molaei *et al*., 2010). Here, despite a relatively small sample size, we found that Galápagos *C. quinquefasciatus* took bloodmeals from a wide range of species. While it predominantly fed on mammals (humans and domesticated), it also fed on native birds and reptiles (7% of cases in the highlands and 23% in the lowlands). Thus, all three taxonomic groups could be exposed to WNV should this pathogen arrive on the islands, including endemic species. This also affirms the propensity of *C. quinquefasciatus* to act as a ‘bridge vector’ for WNV, enabling it to spread beyond an avian‐vector enzootic cycle (Kilpatrick *et al*., 2006a). The propensity of Galápagos *C. quinquefasciatus* to feed on human beings has potential implications for public health in the event of a WNV outbreak on the archipelago. To help inform disease risk assessments, future work should examine if vector feeding is in proportion to relative host abundance or if some host species are preferentially targeted. An initial estimation of foraging indices (calculated as the fraction of bloodmeals from vertebrate species *i*, divided by the relative density of that host species *j* with the vertebrate community) indicates that humans and chickens in the Galápagos are preferentially fed on by *C. quinquefasciatus*, with an underutilization or aversion to feeding on *Geospiza* spp. (Eastwood, unpublished data). This should be kept in mind when addressing the question of the relative importance of candidate WNV vectors on Galápagos, since an avian amplification host is likely required. *Culex quinquefasciatus* would need to take sufficient bloodmeals from competent hosts in order to maintain the WNV if the virus were introduced. Given its better adaptation to brackish water habitats, near ubiquitous distribution and high WNV vector competence, *A. taeniorhynchus* may play an equal or greater role in WNV establishment and transmission (Eastwood *et al*., 2013). However, Galápagos *A. taeniorhynchus* has also been found to feed infrequently on birds (Bataille *et al*., 2012; Eastwood *et al*., 2013), although occasional bird bloodmeals have been detected elsewhere (e.g. heron in Puerto Rico [preference and amplification role unknown]; Barrera *et al*., 2011).

The time from a mosquito taking a bloodmeal to its first deposition of eggs influences generation time and frequency of feeding and, as such, is a critical determinant of pathogen transmission rate and disease dynamics (Kilpatrick *et al*., 2006b; Lardeux *et al*., 2008). As is the case for a variety of mosquito species (Lardeux *et al*., 2008; Mala *et al*., 2014), we found the duration of the gonotrophic cycle for Galápagos *C. quinquefasciatus* to be temperature dependent, but to not be affected by the WNV status of blood consumed. It is possible that bloodmeal host type could influence gonadotrophic cycle duration, yet such an investigation was beyond the scope of this study. In the U.S.A., longer‐lived mosquitoes with shorter gonotrophic cycles are considered to be of greater epidemiological importance for the transmission dynamics of a pathogen, such as WNV, due to increased opportunities for host contact. Our data on gonotrophic cycle length and longevity could be used to estimate 3.8 vector–host contacts per female mosquito life at a mean temperature of 26 °C (based on a bloodmeal being sought every 5.4 days, plus 1 day for blood seeking, over a 24.03 days average lifespan). Future studies should examine biting rates of this species to further inform vectorial capacity. Although Ciota *et al*. (2014) show accelerated immature mosquito development with increasing temperature, they also found reduced adult longevity in field‐collected U.S. *C. quinquefasciatus* (Ciota *et al*., 2014); therefore, seasonal fluctuations in the Galápagos climate may influence vector–host contact. The mean duration of the Galápagos *C. quinquefasciatus* egg–adult lifecycle (10.54 days based on 2.9 ppt salinity) is similar to that of U.S. *C. quinquefasciatus* (10.52 days) held at the same temperature of 25 °C (Rueda *et al*., 1990).

Although earlier and later incubation periods were not assessed, we could detect WNV from the salivary secretions of infected Galápagos *C. quinquefasciatus* between 7 and 28 days post‐infection at titres (averaging 4.1 log_10_ PFU) sufficient to infect a vertebrate host (Styer *et al*., 2007). Whether or not non‐avian hosts in Galápagos (more commonly utilized for mosquito feeding) are likely to acquire high enough titres of WNV to act as amplifying hosts remains a question for further research, e.g. by host‐competency studies of WNV using select Galápagos species.

Vertical transmission has been demonstrated for several arboviruses in a range of mosquito species, and this type of transmission is considered to be underappreciated for flaviviruses (Nayar *et al*., 1986; Miller *et al*., 2000). In the U.S.A., field‐collected *C. quinquefasciatus* larvae have tested positive for WNV RNA (Unlu *et al*., 2010). Goddard *et al*. (2003) provided experimental evidence that *C. quinquefasciatus* can transmit WNV vertically, reporting MFIR of approximately 3/1000 for strains of this mosquito in California, U.S.A. Expanding on our previous study (Eastwood *et al*., 2011), we now detect a similar rate of vertical transmission (MFIR 3.7/1000) in Galápagos *C. quinquefasciatus*. This indicates a potential mechanism for WNV persistence in the area.

In combination, our results can assist future assessments of WNV and other disease agents vectored by *C. quinquefasciatus* in Galápagos. They indicate that this mosquito is likely to be an important (bridge) vector for WNV transmission, should the pathogen reach the archipelago. *Culex quinquefasciatus* is a recent invader of the Galápagos Islands, but has the ecological characteristics to facilitate the spread and persistence of this emerging infectious disease, and possibly others. To limit the range and abundance of this invasive species, we recommend that human‐created fresh water sources are limited and closed to mosquitoes as much as possible. It is important to continue monitoring the distribution and abundance of this species across the Galápagos, including in uninhabited areas, in order to inform effective vector control as necessary.

## Ethics Approval and Consent to Participate

This research was evaluated and approved by the Zoological Society of London's Ethics Committee (ref. WLE477), and the work was conducted under permit granted by the Galápagos National Park (PNG09‐21).

## Author contributions

All authors contributed to the study design. GE implemented field collections, ecological experiments, virology studies and conducted the data analysis, together with the advice of other authors. SJG, LK and AC reviewed and edited the manuscript. All authors read and approved the final version of the manuscript.

## Supporting information


**Figure S1.** Host feeding preferences of *Culex quinquefasciatus* in Galápagos at: (a) Highland sites, (b) Coastal sites.
