# Supplementary material for: The vector ecology of introduced Culex quinquefasciatus populations, and implications for future risk of West Nile virus emergence in the Galápagos archipelago
Source: Med Vet Entomol. 2018 Aug 31;33(1):44–55. doi: 10.1111/mve.12329 (PMC7379259; doi:10.1111/mve.12329)

Figure S1: Host feeding preferences of *Culex quinquefasciatus* in Galápagos at: a) Highland sites, b) Coastal sites.

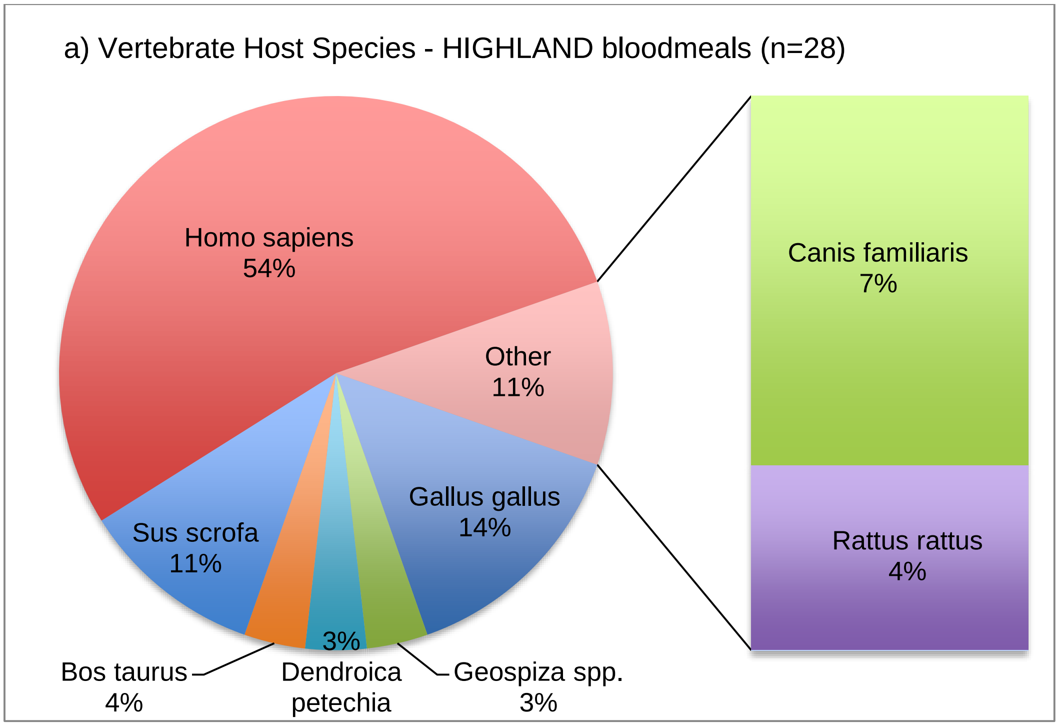

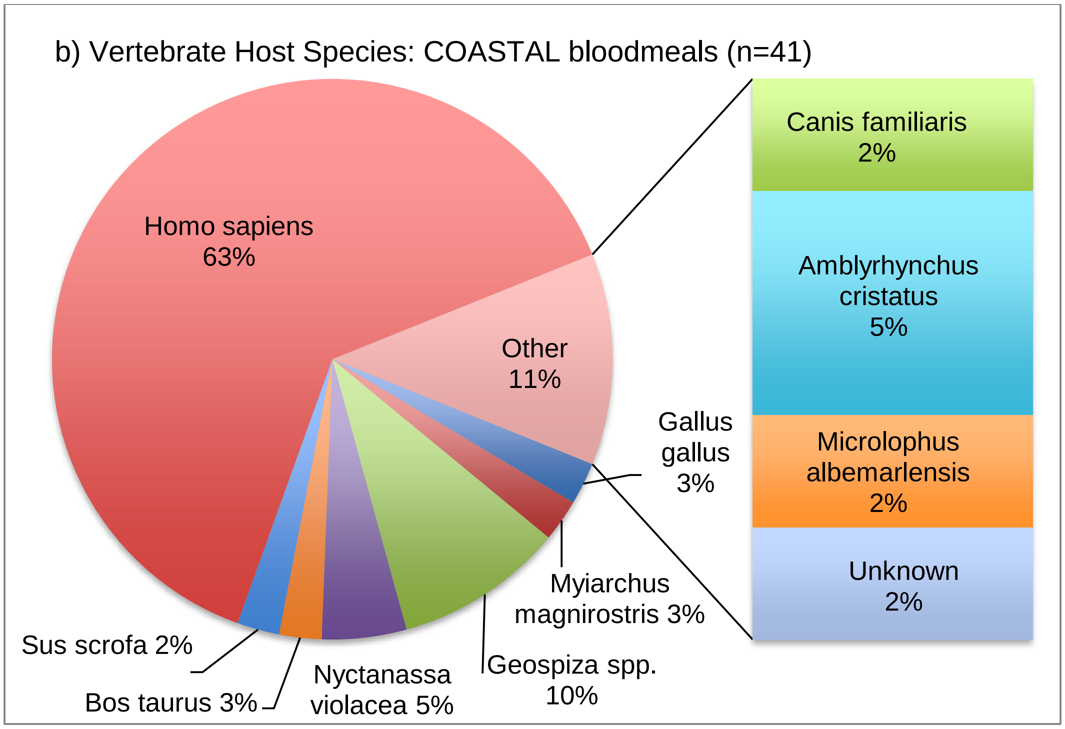

Supplement: Supplementary file 1 — Figure S1. Host feeding preferences of Culex quinquefasciatus in Galápagos at: (a) Highland sites, (b) Coastal sites. [file MVE-33-44-s001.pdf]
